# Integrative analysis of a cancer somatic mutome

**DOI:** 10.1186/1476-4598-6-13

**Published:** 2007-02-05

**Authors:** Pilar Hernández, Xavier Solé, Joan Valls, Víctor Moreno, Gabriel Capellá, Ander Urruticoechea, Miguel Angel Pujana

**Affiliations:** 1 Bioinformatics and Biostatistics Unit, and Translational Research Laboratory, Catalan Institute of Oncology, IDIBELL, L'Hospitalet, Barcelona 08907, Spain

## Abstract

**Background:**

The consecutive acquisition of genetic alterations characterizes neoplastic processes. As a consequence of these alterations, molecular interactions are reprogrammed in the context of highly connected and regulated cellular networks. The recent identification of the collection of somatically mutated genes in breast tumors (breast cancer somatic "mutome") allows the comprehensive study of its function and organization in complex networks.

**Results:**

We analyzed functional genomic data (loss of heterozygosity, copy number variation and gene expression in breast tumors) and protein binary interactions from public repositories to identify potential novel components of neoplastic processes, the functional relationships between them, and to examine their coordinated function in breast cancer pathogenesis. This analysis identified candidate tumor suppressors and oncogenes, and new genes whose expression level predicts survival rate in breast cancer patients. Mutome network modeling using different types of pathological and healthy functional relationships unveils functional modules significantly enriched in genes or proteins (genes/proteins) with related biological process Gene Ontology terms and containing known breast cancer-related genes/proteins.

**Conclusion:**

This study presents a comprehensive analysis of the breast somatic mutome, highlighting those genes with a higher probability of playing a determinant role in tumorigenesis and better defining molecular interactions related to the neoplastic process.

## Background

Recent landmark work has described the genetic landscape of the breast and colorectal cancer genomes by identifying the collection of somatically mutated genes (cancer somatic mutome) that contributes to the neoplastic process in these cancer types [1]. Most of these genes were not previously identified as linked to human cancer and some of them encode uncharacterized proteins. A larger set of "passenger" mutations or mutations present at a frequency that is too low to determine their relationship with cancer were also identified, prompting further genetic and molecular characterization.

Most biological processes involve groups of genes and proteins that behave in a coordinated way to perform a cellular function [2]. The coordinated task of genes/proteins can be represented by different types of functional relationships (e.g. gene co-expression, genetic interactions, protein binary interactions, protein complex membership) [3]. Network modeling has been used to predict new gene/protein functions and to define pathway components or modulators of particular processes [reviewed in [4-6]]. The application of similar approaches has also identified new genes responsible for human diseases [7,8].

Defining biological processes at the systems-level will help to understand cancer cellular networks. The application of an integrative "omic" approach to the breast cancer somatic mutome is encouraged by the identification of uncharacterized genes/proteins and because the complete wiring diagram of functional associations has yet to be determined. The aim of this study is therefore to comprehensively describe the status of candidate breast cancer tumor suppressors and oncogenes at different molecular levels (from gene to protein), to predict new functional relationships between them and to provide new hypotheses regarding their coordinated molecular function in the neoplastic process. This study is focused on the somatic mutome described by Sjoblom et al. [1], which contains validated (contributing to the neoplastic process) and non-validated (i.e. harboring putative "passenger" mutations or mutations present at a frequency that is too low to determine their relationship with the neoplastic process) gene sets (total 672), combined with previously known somatically mutated breast cancer genes compiled in the COSMIC database [9].

## Results

### Loss of heterozigosity analysis

To investigate the role of somatically mutated breast cancer genes as classical tumor suppressors or oncogenes, we first examined genomic loss of heterozigosity (LOH) using a whole-genome SNP genotyping data set [10]. This data set has a resolution of one SNP every ~210 genomic kilo-bases and contains information from 42 breast tumors (20 non basal-like, 18 basal-like and 4 BRCA1 tumors) and matched healthy breast tissue samples.

When all breast tumors were considered, mutated genes in the validated set showed LOH ranging from 4% to a maximum of 76% (*TP53*)(Additional file 1). As was expected, other genes showing relatively high percentages of LOH in breast tumors were *BRCA1 *(52%) and *MRE11A *(50%). Remarkably, of the validated genes only *CDH5 *was previously described in detail as showing LOH [11], which might be explained by the unbiased approach used to identify the breast cancer somatic mutome, or by the inexistence of LOH as a second-hit genetic mechanism common to this set of genes. The detection of ~33% of LOH at the *TMPRSS6 *locus supports its role as a tumor suppressor suggested by a previous observation that *TMPRSS6 *nucleotide variants conferred a risk of breast cancer [12]. However, LOH should be interpreted with caution as it shows a high correlation with chromosome location (e.g. complete LOH of chromosome 17). LOH results do not significantly vary between basal-like and non basal-like tumor subtypes except for the isodisomy of chromosomes 14, 17 and X [10].

**Figure 1:**
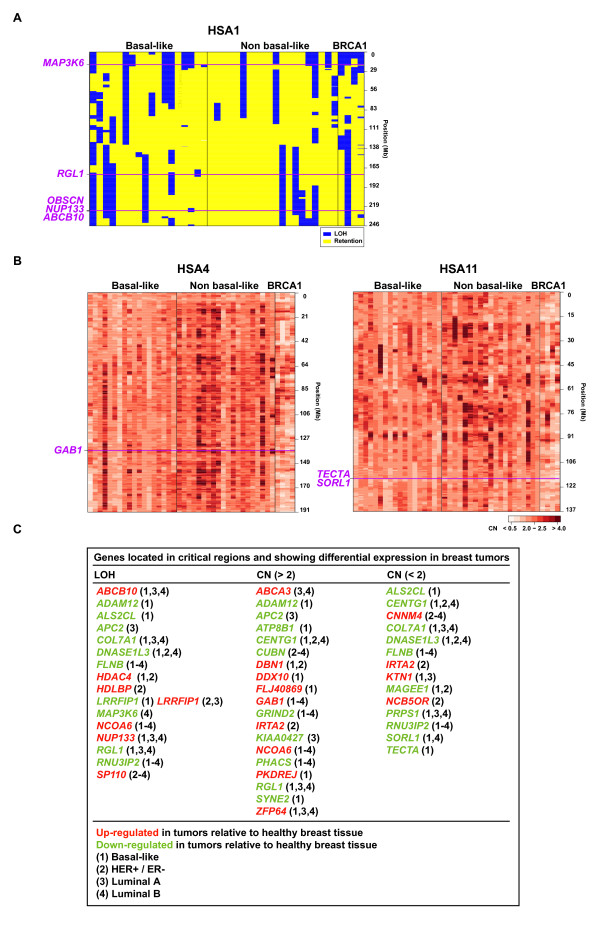
Integration of LOH, CN and expression data to better define candidate tumor suppressors and oncogenes for the breast cancer neoplastic process. Examples of LOH and CN analyses: (A) LOH analysis for HSA1 shows three critical regions (defined by close boundaries of LOH) indicated by pink lines across tumor samples; (B) CN analyses indicate *GAB1 *locus genomic amplification in HSA4, and *SORL1 *and *TECTA *loci genomic loss in HSA11; and (C) Integration of LOH and CN, and differential expression in tumors relative to healthy tissues indicate candidate tumor suppressors (down-regulated in tumors, green) and oncogenes (up-regulated in tumors, red) in four different types of breast tumors as indicated by numbers in brackets.

For a comprehensive understanding of LOH results, we integrated gene expression data available for the same healthy and tumor samples used for SNP genotyping, and combined it with a larger expression data set containing basal-like and other tumor subtypes [13] (Fig. 1). Approximately 50% of mutome genes showed differential expression between healthy and tumor tissue samples. Careful examination of LOH identified 20 genes in the validated set mapping to 12 critical regions (relatively close genomic boundaries of LOH). Expression analysis supports the supposition that 10 of these genes may act as tumor suppressors, as they show down-regulation in breast tumors (Fig. 1C, LOH column and down-regulated genes in tumors). In addition to these genes, a few others showed concordant results between LOH and expression analyses but cannot be mapped to critical regions (*CENTG1, MAGEE1, PRPS1, SYNE2 *and *TP53*). Although not completely clear from LOH, the integration of expression data also supports the role of *ICAM5 *as a tumor suppressor proposed by the identification of nucleotide variants that confer a risk of breast cancer [14]. The present LOH analysis suggests the loss of the *ICAM5 *locus in non basal-like tumors (15%) but not in BRCA1 or basal-like (< 5%) tumors, and its expression appears significantly down-regulated in three distinct types of tumors when compared to healthy tissues [luminal A, luminal B and tumors showing human epidermal growth factor receptor 2 positivity (HER-2+) and estrogen receptor negativity (ER-)]. Collectively, the integration of LOH and expression analyses suggests the hypothesis of the existence of at least ~10 tumor suppressor genes in the breast cancer somatic mutome.

### Copy number analysis

Using the same data set described above, genes in the validated set showed copy numbers (CNs) ranging from 1.60 to 3.37 across basal-like and non basal-like tumors (Additional file 2). As expected for tumors with relatively higher levels of genomic instability, broader margins of CN variation were observed in BRCA1 tumors, ranging from 0.57 to 3.82. Examination of gene expression and critical regions with CN > 2 identified nine candidate oncogenes (Fig. 1C, CN > 2 column and up-regulated genes in tumors). Notably, one of these genes, *GAB1*, was previously suggested to act as an oncogene in cellular transformation [15]. CN analysis also identified critical regions of genomic loss that were not evident in the LOH analysis, such as the *SORL1 *and *TECTA *loci that showed loss and expression down-regulation particularly in basal-like tumors (Fig. 1B and 1C). Thus, eight additional genes showed CN < 2 in a critical region and concordant down-regulation in tumors, which suggests their role as tumor suppressors (Fig. 1C, CN < 2 column and down-regulated genes in tumors).

In addition to the particular genes mentioned above, the correlation of LOH, CN and expression data identified four concordant gene clusters (i.e. close located loci). First, the amplification and over-expression of *ABCB10 *and *NUP133 *genes at chromosome 1 in basal-like and luminal A and B tumors. Remarkably, the amplification of *ATP-binding cassette (ABC) transporter *genes is commonly found in cancer cell lines as a probable mechanism of drug resistance [16] and nuclear pore (NUP) subunits have been found over-expressed in breast tumors [17]. Second, the loss and down-regulation of *COL7A1*, *DNASE1L3*, *FLNB *and *RNU3IP2 *at chromosome 3, particularly in basal-like and luminal B tumors. Third, the loss and down-regulation of *AEGP, GSN, NUP214 *and *SPTAN1 *at chromosome 9, particularly in luminal A and B tumors. Finally, the loss and down-regulation of *SORL1 *and *TECTA *at chromosome 11, particularly in basal-like tumors. These genomic mutome clusters suggest that, in addition to point mutations, large-scale alterations of these regions might constitute a mechanism contributing to the neoplastic process.

### Expression analysis

**Figure 2:**
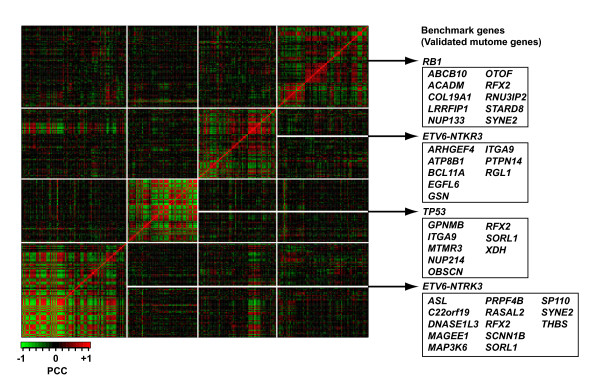
Gene co-expression analysis in breast tumors. Clustering of microarray probes (297 × 297) representing mutome (validated and non-validated) [1] and benchmark (literature) [9] genes according to absolute PCC values. Clusters are named according to the benchmark(s) gene(s) present in each of them (i.e. *RB1*, *ETV6-NTKR3 *or *TP53*-related). Boxes contain validated mutome genes present in each cluster. Non-validated gene names are not shown.

To further determine the level of functional association among somatically mutated breast cancer genes, we investigated their co-expression in a large breast tumor data set containing 98 primary tumors [18]. A total of 878 probes corresponding to 680 (mutome plus benchmark) genes gave rise to 385,003 pair-wise comparisons. A higher number of these pairs than expected by chance show significant co-expression measured by the Pearson's correlation coefficient (PCC) (15,994 significant pairs applying a false discovery rate (FDR) of 0.01). Considering absolute PCC values, four clusters of high expression correlation were observed (Fig. 2). According to the presence of benchmark genes, co-expression clusters could be classified as *ETV6-NTKR3*, *TP53 *or *RB1*-related. Since gene pairs that encode functionally related proteins tend to show higher expression correlation than pairs of unrelated genes, functional associations can be predicted based on profiling comparison. Thus, two genes in the *RB1*-related cluster encode known physical interactors of pRb (*ATF2 *and *CUTL1*, included in the non-validated set) [19,20]. Similarly, the presence of *ABCB10 *and *NUP133*, and candidate tumor suppressors *LRRFIP1 *and *RNU3IP2 *in the *RB1*-related cluster, further support their functional association in breast cancer.

**Figure 3:**
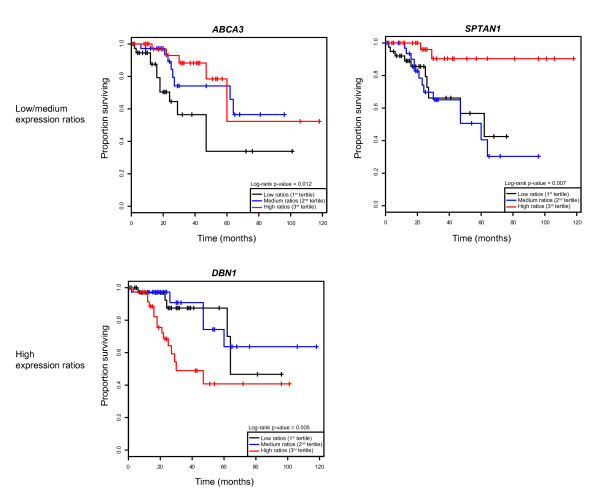
Gene expression analysis and breast cancer survival. Kaplan-Meier survival curves based upon categorized expression in tertiles are shown for three validated genes in the Hu et al. [13] data set.

Next, we examined whether gene expression levels have prognostic value and how this correlates with genomic and expression alterations in breast tumors. We used a data set containing information from 113 patients [13] and performed Kaplan-Meier analyses using the Cox-Mantel log-rank test. Cox's regression models were adjusted and non-adjusted for tumor grade and ER status. This analysis identified four validated genes whose expression levels predict survival (non-adjusted *P *values < 0.001 and adjusted *P *values < 0.05; genes *ABCA3, DBN1, SP110 *and *SPTAN1 *with adjusted hazard ratios (HR) of 0.58, 2.86, 0.59 and 0.20, respectively) (Fig. 3). Two other validated genes were identified with a lower significance level (non-adjusted *P *values < 0.01 and adjusted *P *values < 0.1; *C22orf19 *and *RASGRF2 *withHR of 2.29 and 0.36, respectively) and 17 genes in the non-validated set show association (adjusted *P *values < 0.05) (Additional file 3). Analysis of an independent data set containing information from 295 patients [21] supports the observation that high expression ratios of *DBN1 *predict poor survival (adjusted *P *value of 0.03 and HR of 3.81) and indicates the same tendency as previously noted for low expression ratios of *ABCA3, SP110 *and *SPTAN1 *(non-adjusted HR of 0.31, 0.34 and 0.64, respectively), although this now appears non-significant when adjusted for tumor grade and ER status (adjusted HR of 0.61, 0.25 and 1.19). In the non-validated set, only *WFDC1 *expression remained associated with survival in the multivariate analysis of the independent data (adjusted *P *values of 0.001 and 0.03, and HR of 3.99 and 7.63 for two different microarray probes).

### Interactome analysis

**Figure 4:**
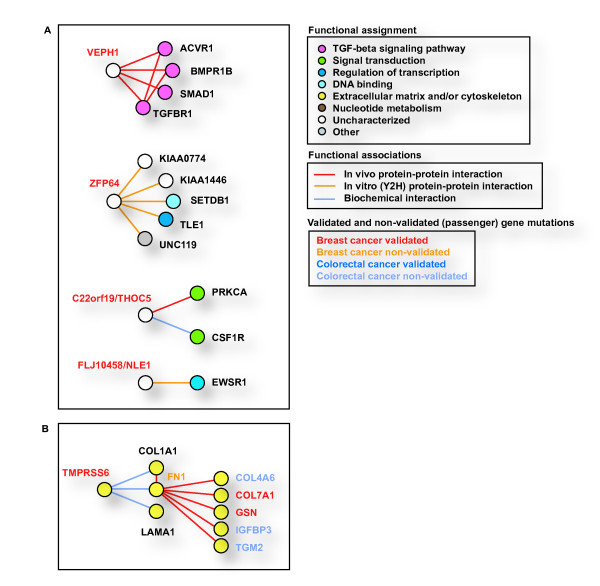
Human interactome network analysis, functional prediction and breast and colorectal cancer mutome association. (A) Predicted interactions for uncharacterized validated mutome gene products. Functional assignment is based on GO term annotations. Protein interactions and node types are indicated as shown in the insets. (B) Breast and colorectal cancer mutome association through extracellular matrix and cytoskeleton constituents.

To evaluate functional associations between proteins, we mapped mutome gene products on the human interactome network [22-24]. Since similar Gene Ontology (GO) annotations are more likely to be present in pairs of interacting proteins than in pairs of unrelated proteins, functional predictions can be formulated based on annotations of neighbor proteins in the network. In particular, the examination of GO annotations provides functional assignment of uncharacterized gene products (Fig. 4A), such as the VEPH1 protein that was identified in a large-scale interactome mapping study of the TGF-beta signaling pathway [25].

An examination of binary protein interactions also highlights the possible need for more detailed mutational analyses of specific cellular components. Thus, an association between the breast and colorectal mutomes identified by Sjoblom et al. [1] is revealed by examining interactions between proteins of the extracellular matrix and cytoskeleton functional module (Fig. 4B). In this module, four out of nine proteins included were found to be mutated in breast tumors and three were found to be mutated in colorectal tumors by Sjoblom et al. [1].

Next, we investigated the existence of coordinated molecular tasks by examining the level of connectivity between mutome gene products in the interactome network. We compared the size (number of nodes and edges) of the largest component generated by direct interactions between mutome validated proteins and compared it to equivalent randomly selected sets of 100 proteins. The results showed that mutome gene products are highly connected, more so than expected by chance (interactions/node, empirical *P *value < 0.05), thus supporting the theory that they are involved in related molecular pathways or functions. However, this observation is partially dependent on the presence of p53 and BRCA1, which exhibit extremely high connectivity. Without taking into account p53 and BRCA1, the level of connectivity of the validated mutome is still moderately high with respect to equivalent, randomly selected protein sets (empirical *P *value < 0.15). These results suggest greater centrality of the breast somatic mutome proteins and are consistent with earlier observations involving previously known human cancer proteins [26].

**Figure 5:**
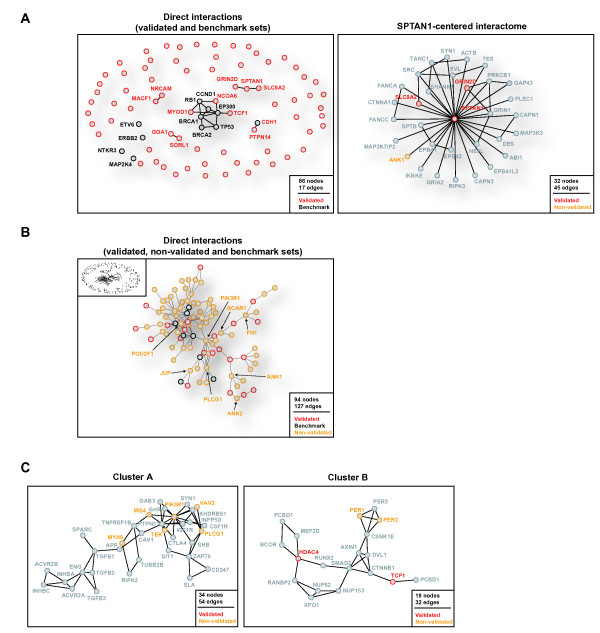
Human interactome network analysis, direct interactions between mutome gene products. (A) Left panel, direct interactions between validated mutome and/or benchmark gene products. Right panel, interactions centered on SPTAN1, whose expression level predicts survival (Fig. 3). Grey nodes represent non-mutome/benchmark proteins. (B) Network generated by direct protein interactions between validated and non-validated mutome and/or benchmark gene products (top left inset). An image of the largest component of this network is shown, with critical nodes that connect benchmark or mutome proteins indicated by arrows. (C) Clusters or densely connected regions in the interactome network that contain more mutome gene products than expected by chance: cluster A shows enrichment in annotations of the TGF-beta and insulin signaling pathways and of DNA transcriptional activity; cluster B shows enrichment for centrosome-related tasks and DNA transcriptional activity.

When only direct interactions are considered between validated and benchmark gene products, examination of the largest network component supports a critical role for three transcription factors or co-activators: MYOD1, NCOA6 and TCF1. These proteins appear included in a module with high connectivity that contains five members of the benchmark set (Fig. 5A). Notably among these genes, *NCOA6 *maps to a critical region of CN > 2 (Fig. 1C). This gene was previously identified as amplified in breast tumors [27] and in this study appeared particularly over-expressed in basal-like tumors.

When non-validated gene products are included in the interactome analysis, a large component with 127 edges and 94 nodes is revealed (Fig. 5B). Eight non-validated gene products occupy critical positions in this component, connecting validated and/or benchmark proteins: BCAR1 (breast cancer anti-estrogen resistance 1) links ADAM12 and GSN, therefore mediating extracellular matrix and cytoskeleton remodeling; and three gene products show a high degree of connectivity (between 5–10 interactions; PIK3R1, PLCG1 and POU2F1), which suggests a central role in the transmission of molecular information within this component. PIK3R1 and PLCG1 are involved in intracellular signaling cascades and their differential regulation is known to be involved in tumorigenesis [28,29], while POU2F1 interacts with several known breast cancer-associated proteins (i.e. BRCA1, BARD1 and PARP1) [30,31]. Together, these observations suggest a coordinated function between validated and non-validated gene products in the breast cancer neoplastic process.

Clustering analysis has previously proved useful for the identification of functionally related genes or proteins [32]. To further examine the higher-level organization of the breast cancer mutome, we identified densely interconnected regions of the interactome harboring a higher proportion of mutome gene products than expected by chance. One such cluster shows enrichment in functional annotations of the TGF-beta and insulin signaling pathways as well as DNA transcriptional activity (Fig. 5C, cluster A). Another cluster shows enrichment for centrosome-related tasks and DNA transcriptional activity (Fig. 5C, cluster B). Cluster enrichment therefore points to known critical functional modules involved in breast tumorigenesis.

### Mutome network modeling

**Figure 6:**
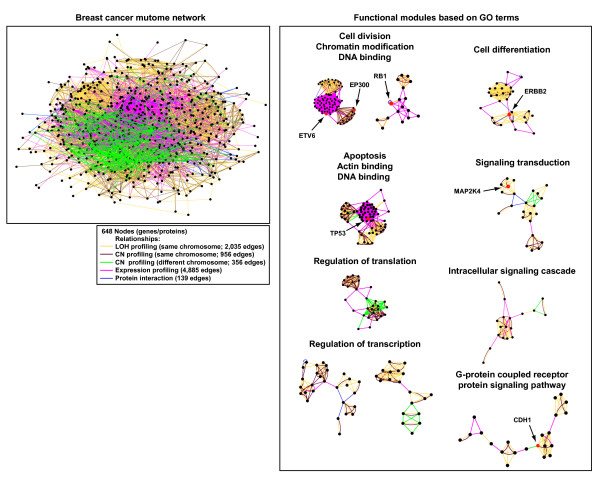
Breast cancer mutome network modeling. Left panel, five functional genomic or proteomic, pathological or healthy-related associations; each one indicated by one of the colored lines shown in the inset was included to generate a mutome network model. Right panel, clusters or densely connected regions in the network that show enrichment in GO terms (functional modules). Benchmark nodes present in these functional modules are marked by arrows.

To generate a network model containing relevant biological information for the breast cancer neoplastic process, we integrated different types of functional relationships identified through the genomic (i.e. LOH, CN and expression) and proteomic (i.e. interactome) analyses explained above. Thus, using network modeling we connected two nodes when their corresponding genes showed similar LOH, CN or expression profiles across breast tumors (see Methods), or when their corresponding encoded gene products were directly connected in the interactome network. The breast cancer mutome network contains 648 nodes and 8,371 edges, and shows a high degree of connectivity that further supports the existence of biologically related functions (Fig. 6 and Additional file 4).

Cluster analysis of this network identifies underlying molecular mechanisms of breast cancer. Analysis of densely connected sub-graphs and their GO terms identified functional modules enriched for apoptosis, cell division, cell differentiation, G-protein coupled receptor protein signaling pathway, intracellular signaling cascade, regulation of transcription, regulation of translation and signaling transduction (Fig. 6). Some benchmark genes/proteins can be located in these modules, supporting their role in the neoplastic process. These observations support the theory that the network modeled here represents a framework for a more in-depth experimental study of genes/proteins related to breast cancer somatic alterations.

## Discussion

Although issues of specificity and sensitivity in the detection of the mutome will probably be addressed in the future, particularly regarding germline genomic CN variation [33] and the likelihood of detecting sequence changes as presented by Sjoblom et al. [1], by examining functional genomic (LOH, CN and gene expression) data in breast tumors, this study supports newly identified tumor suppressors and oncogenes. Through the examination of protein binary interactions, this study further provides new hypotheses regarding the functional associations of these gene products. Finally, the integration of pathological and healthy functional relationships generated a mutome network model that provides a framework for studying the coordinated molecular function of mutome genes/proteins.

The apparent discrepancy between cancer genomic and expression changes for some genes, such as genomic CN > 2 and expression down-regulation, is not exceptional and has been observed previously [34]. Autoregulation of gene expression, dosage compensation, epistatic modifications, or merely issues such as the sensitivity and specificity of LOH/CN and expression analyses can explain these apparent discrepancies. As is to be expected, the proportion of down-regulated genes is higher in CN < 2 than in CN > 2 regions, while the proportion of up-regulated genes is higher in CN > 2 than in CN < 2 regions (Fig. 1C). Nonetheless, experimental investigation of these genes/proteins is required to demonstrate their role as tumor suppressors or oncogenes.

The integrative study also serves as an indication of new prognosis markers. For the mutome genes, the integrative analysis of genomic copy number and expression data strongly indicates that *DBN1 *is a candidate oncogene that, when highly expressed in tumors with respect to healthy tissues, predicts poor survival in breast cancer patients (Fig. 3). Low expression ratios of *ABCA3 *and low or medium expression ratios of *SPTAN1 *may also predict poor survival. *ABCA3 *was previously identified as an ER-regulated gene [35], which supports its involvement in breast tumorigenesis, and *SPTAN1 *was involved in chemotherapy resistance in ovarian cancer [36], which makes this gene a potential target for cancer treatment. Finally, the interactome analysis of molecular pathways provides new hypotheses for the identification of genes potentially associated with survival outcome. SPTAN1 interacts with GRIND2 and SLC9A2, both of which interact with the product of the *ABL1 *proto-oncogene. Activated ABL1 kinase promotes invasion of breast cancer cells [37]. Since low expression ratios of *SPTAN1 *predict poor survival, SPTAN1 could therefore act as a negative regulator of ABL1 activity.

The integration of omic data highlights likely functional candidates of a particular biological process with increased confidence [7,38]. The strategy used here is applicable to other cancer types and would help to identify new tumor suppressor genes and oncogenes and the wiring diagram of functional interactions between them. The analysis of the breast cancer somatic mutome indicates that at least a few of the genes identified by Sjoblom et al. [1] play a key role in the breast cancer neoplastic process. These results will help to focus subsequent experimental characterizations on key gene/protein candidates.

## Conclusion

We have presented the first comprehensive omic analysis of a cancer somatic mutome. Our analysis supports the theory that a few of these genes play a key role in the breast cancer neoplastic process. This study also provides new hypotheses for the coordinated function of these genes/proteins as tumor suppressors or oncogenes. Network modeling identifies hundreds of new potential pathological associations between the cancer genes/proteins studied. Extensive future research will be carried out by different groups focusing on each of the candidate genes highlighted by Sjoblom et al. [1]. Our study provides a possible framework for the appropriate initial categorization of these genes.

## Methods

### Genomic data analysis

To analyze LOH and CN alterations in breast tumors, we used the Gene Expression Omnibus (GEO) record GSE3743 [10]. Data were normalized and modelled using dChip software [39]. LOH and CN were obtained after mapping genes in build 35.1 of the NCBI human genome sequence. For each gene and sample we took the closest SNPs to infer LOH and CN. If there was a mismatch in LOH calling for surrounding SNPs, the gene was left as missing for that particular sample. LOH profile correlation and confidence intervals (CI) were computed using Cohen's kappa coefficient of agreement, suitable for categorical data. We then classified genes as showing similar profiling if the lower limit of the CI was greater than 0.6. PCC was used to assess CN profile correlations, setting 0.6 as the lower cut-off. To determine the level of correlation between gene expression and genomic CN variation, we used PCC and FDR adjusted *P *values. All these analyses were performed using the R statistical software package [40].

### Gene expression data analysis

Breast cancer gene expression was analyzed using two large data sets [10,13]. Data from Richardson et al. [10] was down-loaded from the GEO record GSE3744 and analyzed using the limma and affy packages in R. Background correction, normalization and averaging of expression values were computed using the RMA algorithm [41]. Differentially expressed genes were detected after computing an empirical Bayes moderated t-statistic and *P *values adjusted by a FDR of 5%. Data obtained from Hu et al. [13] was previously normalized and analyzed using the *t-*test. To evaluate co-expression, we used the data set of van 't Veer et al. [18], calculated PCCs and significance levels based on the t-distribution. A hierarchical algorithm was used to cluster genes, taking as distance the absolute value of 1-PCC. To evaluate prognosis, we used the Hu et al. data set [13] and fitted a Cox regression model to each gene using the overall survival information. An adjusted model taking into account tumor grade and ER status was also fitted for each gene. Likelihood ratio tests were used to evaluate the effect of gene expression on survival. For genes that appeared significant in both models, expression was categorized into tertiles using Kaplan-Meier curves. For these genes, the (non-parametric) log-rank test was calculated. The replica data set used for survival analysis was that of Chang et al. [21].

### Human interactome network and clustering analyses

The human interactome network was built by combining three previously published data sets, which mainly represent experimentally-verified interactions [22-24]. The Gandhi et al. [22] data set contains compiled and filtered protein binary interactions from all currently available databases (HPRD, BIND, DIP, MINT, INTACT and MIPS). High-confidence yeast two-hybrid interactions from Rual et al. [24] and Stelzl et al. [23] were then included. After removing common interactions between the three data sets, the resulting network contained 8,174 nodes and 27,810 edges. The Molecular Complex Detection (MCODE) algorithm [42] was used to detect densely connected regions in the interactome network. To calculate the enrichment of mutome proteins in network clusters, a binomial distribution was used. Enrichment in GO terms was investigated using OntoExpress tools [43] and GENECODIS [44]. To determine the level of connectivity between validated mutome gene products, we compared the number of nodes and interactions in the largest component generated by direct interactions between these proteins (73 of 122 were mapped in the interactome) to the number of nodes and interactions generated by 100 iterations of 73 randomly chosen proteins in the interactome.

## Competing interests

The author(s) declare that they have no competing interests.

## Authors' contributions

PH compiled and analyzed the expression and interactome data sets. XS compiled and analyzed genomic loss of heterozygosity and copy number data. JV performed the gene co-expression and survival analyses. PH, XS, JV and AU helped to draft the manuscript. VM and GC provided institutional support and participated in scientific discussions. AU and MAP conceived the study. MAP designed and coordinated the study, and wrote the original and final versions of the manuscript. All authors have read and approved the final version of the manuscript

## Supplementary Material

Additional File 1 LOH analyses results. Click here for file

Additional File 2 CN analyses results. Click here for file

Additional File 3 Cox regression analyses of non-validated mutome genes using the Hu et al. [13] data set. Click here for file

Additional File 4 Functional relationships in the mutome network model. Click here for file
